# Experimental H-type bovine spongiform encephalopathy characterized by plaques and glial- and stellate-type prion protein deposits

**DOI:** 10.1186/1297-9716-42-79

**Published:** 2011-06-23

**Authors:** Hiroyuki Okada, Yoshifumi Iwamaru, Morikazu Imamura, Kentaro Masujin, Yuichi Matsuura, Yoshihisa Shimizu, Kazuo Kasai, Shirou Mohri, Takashi Yokoyama, Stefanie Czub

**Affiliations:** 1 Prion Disease Research Center, National Institute of Animal Health, 3-1-5 Kannondai, Tsukuba, Ibaraki 305-0856, Japan; 2 Canadian and OIE Reference Laboratories for BSE, Canadian Food Inspection Agency Lethbridge Laboratory, Lethbridge, Alberta, Canada

## Abstract

Atypical bovine spongiform encephalopathy (BSE) has recently been identified in Europe, North America, and Japan. It is classified as H-type and L-type BSE according to the molecular mass of the disease-associated prion protein (PrP^Sc^). To investigate the topographical distribution and deposition patterns of immunolabeled PrP^Sc^, H-type BSE isolate was inoculated intracerebrally into cattle. H-type BSE was successfully transmitted to 3 calves, with incubation periods between 500 and 600 days. Moderate to severe spongiform changes were detected in the cerebral and cerebellar cortices, basal ganglia, thalamus, and brainstem. H-type BSE was characterized by the presence of PrP-immunopositive amyloid plaques in the white matter of the cerebrum, basal ganglia, and thalamus. Moreover, intraglial-type immunolabeled PrP^Sc ^was prominent throughout the brain. Stellate-type immunolabeled PrP^Sc ^was conspicuous in the gray matter of the cerebral cortex, basal ganglia, and thalamus, but not in the brainstem. In addition, PrP^Sc ^accumulation was detected in the peripheral nervous tissues, such as trigeminal ganglia, dorsal root ganglia, optic nerve, retina, and neurohypophysis. Cattle are susceptible to H-type BSE with a shorter incubation period, showing distinct and distinguishable phenotypes of PrP^Sc ^accumulation.

## Introduction

Bovine spongiform encephalopathy (BSE), which belongs to a group of diseases called transmissible spongiform encephalopathies (TSE), is a fatal neurodegenerative disorder of cattle. BSE was first identified in the United Kingdom in 1986 [1], then spread to European as well as North American countries and Japan, and has affected more than 190 000 cattle in the world. The infectious agent responsible for TSE is the disease-associated prion protein (PrP^Sc^), which is thought to be a post-translationally modified form of the host-encoded membrane glycoprotein (PrP^C^) [2]. According to the protein-only hypothesis, PrP^Sc ^is the principal component of the infectious agent.

On the basis of uniform pathology and biochemical profile of the protease-resistant prion protein (PrP^res^) among BSE-affected cattle, it is assumed that BSE in cattle is caused by only one prion strain. Since 2003, variants of BSE (named atypical BSE) have been detected in Japan, Europe, and North America and classified in at least two groups, namely, H-type and L-type BSE, according to the molecular mass of PrP^res^, compared with those of the classical BSE (named C-type BSE) [3]. H-type BSE was first identified in France [4], and L-type BSE, called bovine amyloidotic spongiform encephalopathy (BASE), was first detected in Italy [5]. It is accepted that C-type BSE is caused by the consumption of BSE-contaminated feed, whereas the origins of H-type and L-type BSE remain enigmatic. Hypotheses for the origin of atypical BSE include (1) infection of cattle with different BSE agents; (2) infection of cattle with a non-bovine source or unrecognized forms of infectious TSE agents; (3) genetic mutations in the prion protein gene; and (4) spontaneous or so-called sporadic forms of TSE in cattle, limited to old age, like the sporadic form of human Creutzfeldt-Jakob disease (CJD) [6-10]. However, only one genetic mutation has been found in an H-type BSE case [11]. Sequence analysis of the open reading frame (ORF) of the prion protein gene (*PRNP*) has not revealed any mutations in atypical BSE cases in France [4], Italy [5], and Canada [12]. Therefore, it seems unrealistic to suggest a genetic origin of atypical BSE [13]. The transmissibility of atypical H-type and L-type BSE to mice [13-18] and cattle [19-22] has been confirmed, and these forms clearly differ from C-type BSE regarding incubation periods, PrP^res ^profiles, protease susceptibility, and spatial distribution patterns of histopathological lesions and immunolabeled PrP^Sc ^[3,6,16,20,22]. Interestingly, C-type [23] and H-type [14,15] BSE isolates were transmissible to wild-type mice already in the first passage, whereas L-type BSE agent failed to transmit in the first passage but was successfully transmitted to wild-type mice in the second passage [17].

Unfortunately, a detailed and all-encompassing analysis of neuropathology and topographical distribution of immunolabeled PrP^Sc ^in H-type BSE-affected cattle could not be performed, since only the obex region is routinely sampled for BSE surveillance testing and the remaining brain as well as the carcasses are not available in most countries [3,10,12,13,24-27]. Recently, clinical signs and biochemical properties of experimental German H-type BSE cases have been reported [20]. The primary objective of this study was to investigate the transmissibility of H-type BSE, using a field isolate detected in the active surveillance program in Canada [12]. The secondary objective was to extend the knowledge of the topographical distribution and deposition patterns of immunolabeled PrP^Sc ^in H-type BSE.

## Materials and methods

### Animal inoculation

All animal experiments were approved by the Animal Ethical Committee and the Animal Care and Use Committee of National Institute of Animal Health. The Canadian H-type BSE case was of a Charolais cross displaying signs of recumbency prior to euthanasia [12]. By confirmatory immunohistochemistry, the staining pattern was characterized by a predominant reaction in the neuropil (including glial cells) and a relatively low level of intraneuronal, particulate, and stellate immunolabeling in the obex region. The molecular features of PrP^Sc ^in cattle were described for the Canadian H-type BSE [12] and C-type BSE [21]. Brain homogenates were prepared in nine volumes of phosphate-buffered saline (PBS, pH 7.4), using a multibead shocker (Yasui Kikai Co., Osaka, Japan). Two female and one neutered 3- to 4-month-old Holstein calves were challenged intracerebrally with 1 mL of the 10% brain homogenate prepared from the H-type BSE case. Intracerebral transmission of C-type BSE has previously been reported in cattle [21]. In brief, the inoculum was injected into the midbrain via an 18-gauge 7-cm-long disposable needle (NIPRO, Osaka, Japan), following which the needle was withdrawn from the brain. Two sham-inoculated Holstein calves served as controls; they were euthanized at the age of 27 months.

### Tissue processing for histology

The left brain, including the brainstem and cerebellum, of the H-type BSE case was fixed in 10% neutral buffered formalin (pH 7.4), while the contralateral side was frozen at -80°C for western blot analysis of PrP^Sc^. Tissues of C-type BSE for this study were derived from previously reported experimental cases [21]. Coronal slices of the formalin-fixed sample from each animal were cut serially, treated with 98% formic acid for 60 min at room temperature (RT) [28], embedded in paraffin wax, sectioned at 4-μm thickness, stained with hematoxylin & eosin (HE), and used for immunohistochemistry.

The lesion profile was determined in the HE-stained sections by scoring the vacuolar changes in 17 different brain areas as previously described [29]. Selected brain sections were stained with phenol Congo red [30]. In brief, Congo red dye dissolved at 0.2 g in 100 mL of distilled water was mixed with 9 g of NaCl and subsequently with an equal volume of 100% ethanol. This mixture was allowed to stand on ice for 10 min. After filtration, phenol (Nacalai Tesque, Kyoto, Japan) was added at 5 g in 100 mL of the supernatant, and the pH was adjusted to around 3.0 by adding glacial acetic acid. The sections were stained in this solution for 1 h. After hematoxylin counterstaining, the sections were examined under a polarizing microscope.

### PrP^Sc ^immunolabeling

Dewaxed sections were treated with 3% hydrogen peroxide for 10 min, followed by incubation with 10 μg/mL proteinase K (PK) at RT for 10 min. Thereafter, the sections were subjected to an antigen retrieval protocol by alkaline hydrolysis at 60°C for 10 min in 150 mM sodium hydroxide [31]. They were incubated with 10% normal goat serum for 10 min and then with the following anti-PrP primary antibodies for 60 min: SAF32, SAF54, SAF84, 12F10, F89/160.1.5, F99/97.6.1, T1 [32], 44B1 [33], and 43C5 [33] as the 9 monoclonal antibodies (mAbs) and B103 [34] and T4 [35] as the 2 rabbit polyclonal antibodies (pAbs) (Table 1). Working concentrations of the mAbs and pAbs were 1 μg/mL and 5 μg/mL, respectively. Immunolabeling was performed with an anti-mouse or anti-rabbit universal immunoperoxidase polymer (Nichirei Histofine Simple Stain MAX PO (M) or (R); Nichirei, Tokyo, Japan) for 30 min, and the reaction was visualized using 3,3'-diaminobenzidine tetrachloride as the chromogen for 7 min. Finally, the sections were slightly counterstained with Mayer's hematoxylin. To observe the topographical distribution of PrP^Sc ^in the brain with the naked eye, the immunolabeled sections were photographed and viewed with Microsoft PowerPoint.

**Table 1:** Characteristics of the 11 antibodies and the epitope location of the bovine PrP

Antibodies		Epitope		Clonality	Immunogen	Source**
					
		Location	Type*			
N-terminal region	SAF32	62-92	L	Monoclonal	SAF from infected hamster brain	SPI-Bio (Montigny-le-Bretonneux, France)
	B103	103-121	L	Polyclonal	Cattle recPrP	FUJIREBIO (Tokyo, Japan) [34]
	F89/160.1.5	148-155	L	Monoclonal	Cattle recPrP	VMRD (Pullman, WA, USA)
	T1	149-153	L	Monoclonal	Mouse recPrP	Dr Tagawa
	12F10	154-163	L	Monoclonal	Hamster recPrP	SPI-Bio
Core region	SAF54	168-172	L	Monoclonal	SAF from infected hamster brain	SPI-Bio
	44B1	168-242	DC	Monoclonal	Mouse recPrP	Dr Horiuchi [33]
	SAF84	175-180	L	Monoclonal	SAF from infected hamster brain	SPI-Bio
	43C5	175-181	L	Monoclonal	Mouse recPrP	Dr Horiuchi [33]
C-terminal region	T4	221-239	L	Polyclonal	Cattle recPrP	Dr Sata [35]
	F99/97.6.1	229-235	L	Monoclonal	Cattle recPrP	VMRD

SAF, scrapie-associated fibrils; recPrP, recombinant prion protein

*L, linear; DC, discontinuous

**manufacturer and reference number

### Western blotting

The CNS tissues were homogenized in a buffer containing 100 mM NaCl and 50 mM Tris-HCl (pH 7.6). The homogenate was mixed with an equal volume of detergent buffer containing 4% (w/v) Zwittergent 3-14 (Merck, Darmstadt, Germany), 1% (w/v) Sarkosyl, 100 mM NaCl, and 50 mM Tris-HCl (pH 7.6) and incubated with 0.25-mg collagenase, followed by incubation with PK (final concentration, 40 μg/mL) at 37°C for 30 min. PK digestion was terminated using 2 mM Pefabloc. The sample was then mixed with 2-butanol:methanol (5:1) and centrifuged at 20000 *g *for 10 min.

PrP^Sc ^was extracted from the peripheral nervous, extranervous, and lymphoid tissues by phosphotungstic acid precipitation as described previously [36]. The extracted samples were mixed with a gel-loading buffer containing 2% (w/v) sodium dodecyl sulfate (SDS) and boiled for 5 min before electrophoresis. The samples were then separated by 12% SDS-polyacrylamide gel electrophoresis (PAGE) and electrically blotted onto a polyvinylidene fluoride (PVDF) membrane (Millipore, Billerica, MA, USA). The blotted membrane was incubated with anti-PrP mAbs 6H4 (Prionics, Schlieren, Switzerland) and SAF84 at RT for 60 min. Signals were developed with a chemiluminescent substrate (SuperSignal; Pierce Biotechnology, Rockford, IL, USA). After PK treatment, some samples were deglycosylated with *N*-glycosidase F (PNGase F; New England Biolabs, Beverly, MA, USA), according to the manufacturer's instructions.

### Polymerase chain reaction (PCR) amplification and DNA sequencing

In each case, genomic DNA was isolated and purified from the liver using a GenElute mammalian genomic DNA purification kit (Sigma, St. Louis, MO, USA) according to the manufacturer's instructions. The ORF (792 bp) of *PRNP *was amplified using the primers 5'-ATGGTGAAAAGCCACATAG-3' and 5'-CTATCCTACTATGAGAAAAATG-3'. The purified PCR products of the bovine *PRNP *were directly sequenced using ABI 3100-*Avant *sequencer (Applied Biosystems, Foster City, CA, USA), with the abovementioned PCR primers. The nucleotide sequences obtained for the 3 challenged calves were aligned using the GENETYX software (GENETYX Co., Tokyo, Japan), with the following GenBank accession numbers: AY367641, AY367642, and AY367643.

## Results

### Clinical signs

The 3 challenged calves developed initial signs of clinical disease approximately 12 months post challenge, which included disturbance, anxiety, and occasionally low head carriage. After 3-4 months of the onset of the clinical disease, the animals showed loss of body condition. Around 7-10 days prior to euthanasia, the animals developed ataxia of the forelimbs and hindlimbs and myoclonus and were unable to rise. The cattle were euthanized at 507 (case 1, code 7749), 574 (case 2, code 9458), and 598 (case 3, code 0728) days post challenge (mean ± standard deviation, 559.7 ± 47.2 days). The clinical signs were similar in all the 3 H-type BSE-challenged animals. The animals did not show any change in temperament, such as nervousness or aggression.

### Neuropathology

**Figure 1:**
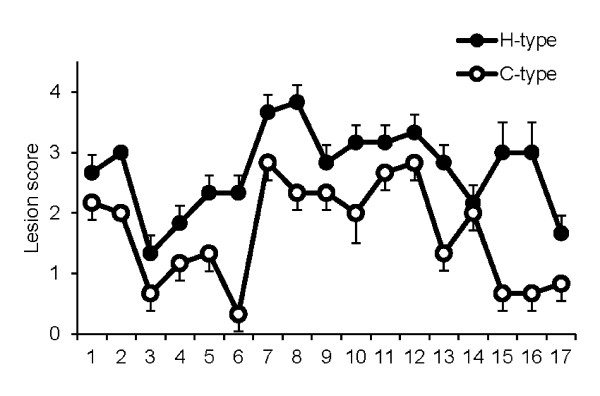
**Lesion profile of H-type BSE-challenged cattle in 17 brain areas**. The different brain areas indicated are as follows: 1, nucleus of the solitary tract; 2, nucleus of the spinal tract of the trigeminal nerve; 3, hypoglossal nucleus; 4, vestibular nuclear complex; 5, cochlear nucleus; 6, cerebellar vermis; 7, central gray matter; 8, superior colliculus; 9, medial geniculate nucleus; 10, hypothalamus; 11, dorsomedial nucleus of the thalamus; 12, ventral intermediate nucleus of the thalamus; 13, frontal cortex; 14, septal nucleus; 15, caudate nucleus; 16, putamen; 17, claustrum. The lesion scores for C-type BSE are taken from a previous study [21].

Scores for the distribution and severity of vacuolation in the brain were similar among the 3 challenged calves. Vacuolar changes were generally observed in all the brain areas. In general, the vacuoles varied in size. The highest mean lesion scores appeared in the thalamic nuclei and neuropil of the central gray matter of the midbrain, and the lowest scores were found in the caudal cerebral and cerebellar cortices. In the vestibular and pontine nuclei, spongy changes were not as prominent as in the other brainstem nuclei. In the spinal cord of the animals with clinical disease, mild vacuolation was present in the neuropil of the gray matter. The detailed vacuolar lesion profile is shown in Figure 1. Lesion scores for C-type BSE in cattle have been previously described [21].

### PrP^Sc ^immunohistochemistry

**Figure 2:**
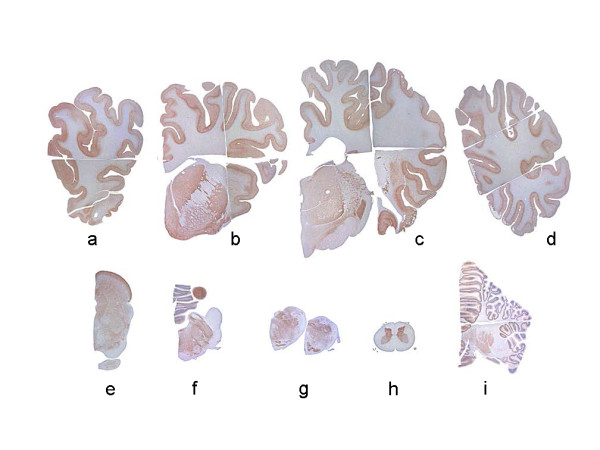
**Topographical distribution of PrP**^**Sc **^**in the CNS of H-type BSE-challenged cattle**. An H-type BSE-challenged case (case 1, code 7749) displays prominent immunolabeling in the cerebral and cerebellar cortices, basal ganglia, thalamus, brainstem, and spinal gray matter, but relatively sparse immunolabeling in the hypothalamus. The 9 different areas indicated are as follows: a, frontal cortex; b, septal nucleus; c, temporal and parietal cortices and thalamus; d, occipital cortex; e, midbrain; f, pons; g, medulla oblongata at the obex; h, spinal cord; i, cerebellum. The sections show immunohistochemical labeling with mAb F99/97.6.1 and hematoxylin counterstaining.

**Figure 3:**
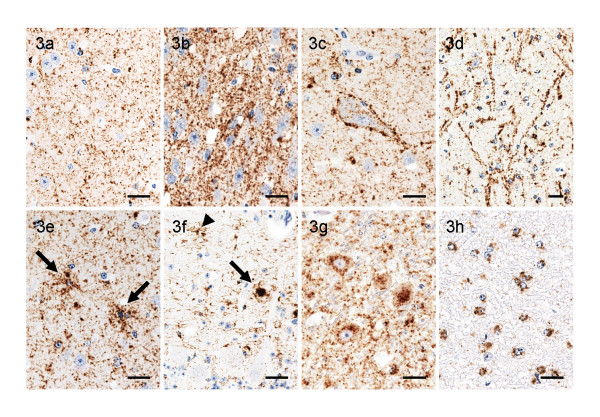
**PrP**^**Sc **^**accumulation patterns in the brain of H-type BSE-challenged cattle**. Fine particulate-type PrP^Sc ^accumulation in the neuropil of the frontal cortex (a). Coarse particulate-type (b), perineuronal-type (c), and linear-type (d) PrP^Sc ^deposition in the thalamus. Stellate-type deposition in the caudate nucleus (e, arrows). Plaque-like (f, arrow) and stellate-type (f, arrowhead) deposition in the cerebral cortex. Intraneuronal-type PrP^Sc ^accumulation in the olivary nucleus (g). Intraglial-type PrP^Sc ^deposition in the cerebellar medulla (h). The images show immunohistochemical labeling with mAb F99/97.6.1 and hematoxylin counterstaining. Scale bars = 20 μm.

**Figure 4:**
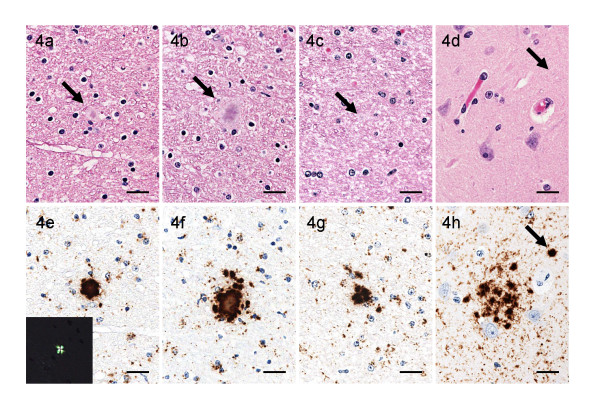
**Various types of plaques**. Various types of plaques stained with HE (a-d) and immunohistochemical labeling with mAb F99/97.6.1 (e-h). Unicentric (a and e) and multicentric (b-d and f-h) cores of plaques (arrows) in the white matter of the thalamus (a-c and e-g) and in the deep cortical area of the frontal lobe (d and h). The inset in the lower left corner of (e) shows an amyloid plaque detected by Congo red staining in the serial section; it shows birefringence under polarized light. Scale bars = 20 μm.

Initially, immunohistochemistry was performed with the C-terminus PrP specific antibody F99/97.6.1. Large amounts of PrP^Sc ^were deposited diffusely in the cerebral cortex, basal ganglia, thalamus, hypothalamus, brainstem, and spinal cord of all three challenged animals (Figure 2). The most conspicuous type of PrP^Sc ^deposition was fine or coarse particulate-type deposition in the neuropil of the gray matter throughout the brain and spinal cord of all the animals (Figures 3a and 3b). Linear, perineuronal, and intraneuronal types of PrP^Sc ^staining, usually detected in C-type BSE-affected cattle, were observed in the cerebral cortex, basal ganglia, thalamus, and brainstem of the H-type BSE-challenged cattle (Figure 3). The deposition pattern of PrP^Sc ^was characterized by the presence of stellate, intraglial, and plaque forms in the brain of the H-type BSE-challenged cattle (Figure 3). Stellate-type PrP^Sc ^deposition was predominantly identified in the cerebral cortex, basal ganglia, thalamus, hypothalamus, and hippocampus and often in the cerebellar cortex, but was not visible in the brainstem and spinal cord (Figure 3e). Intraglial-type PrP^Sc ^deposition was very consistent throughout the white matter of the central nervous system (CNS) and spinal cord (Figures 3h and 4e-h).

PrP^Sc^-positive plaques were scattered throughout the cerebral white matter (Figure 4). Some PrP^Sc^-positive were also detected in the deep cortical layer of the cerebrum, internal capsule of the corpus striatum and thalamic white matter, and cerebellum (Figures 3f and 4), but were absent from the brainstem and spinal cord. On the basis of the morphology of the cores, as revealed after application of immunohistochemistry, the plaques were classified into unicentric and multicentric types (Figure 4). Unicentric plaques had a single core and were up to 25 μm in diameter (Figures 4a and 4e). Multicentric plaques were composed of multiple smaller cores clustered together or a central core surrounded by even smaller plaques or aggregates, varied in shape and could extend up to 40 μm in diameter (Figures 4b-4d and 4f-4h). Both types of plaques were subdivided into two subtypes, that is, those with a dense compact core and others with a pale central core. The dense compact core plaques were less than 20 μm in diameter and smaller than the plaques with a pale central core; they were uniformly immunolabeled and were difficult to detect in HE or Congo red-stained sections (Figures 4c and 4g). Furthermore, plaques with a pale central core were generally larger than that with a dense compact core and stained pale basophilic or amphophilic with HE and positively with Congo red under polarized light (Figure 4e). The periphery of these plaques looked like a halo unstained with HE and Congo red but well immunolabeled with PrP-specific antibodies. In addition, the granular form of plaque-like deposits, was rarely detected in the deep cerebral cortex, basal ganglia, and thalamic nuclei and not detected in the white matter (Figures 4d and 4h). These deposits were not stained with Congo red and were composed of aggregates about 5 μm in diameter.

### Variability of PrP^Sc ^immunolabeling with antibodies

The H-type cases were further investigated by immunohistochemistry also using a set of other PrP specific antibodies covering the different regions of PrP (Table 1). Results of PrP^Sc ^immunolabeling with each antibody are summarized in Table 2. The immunolabeling intensity of each type of PrP^Sc ^varied with the different primary antibodies. The strongest immunolabeling for both extracellular and intracellular PrP^Sc ^was evident with mAb F99/97.6.1 and pAb T4, which recognized the C-terminal region of PrP, whereas mAb SAF32 and pAb B103, which recognized the N-terminal region of PrP, showed no or weak immunolabeling. In general, the core-specific antibodies used in this study produced varying degrees of immunolabeling intensity for each extracellular-type PrP^Sc ^deposit. Intraneuronal-type PrP^Sc ^deposits in H-type BSE showed weaker immunolabeling with the core-specific antibodies than those in C-type BSE. In addition, the core-specific antibodies did not show any immunolabeling for intraglial-type PrP^Sc ^deposits.

**Table 2:** Comparison of immunolabeling intensities of different PrPSc types between C-type and H-type BSE

	**Extracellular PrP**^**Sc **^**type**										**Intracellular PrP**^**Sc **^**type**			
**Antibody**	**Particulate**		**Perinuclear**		**Linear**		**Plaque-like**		**Stellate**		**Intraneuronal**		**Intraglial**	
	
	**C-type**	**H-type**	**C-type**	**H-type**	**C-type**	**H-type**	**C-type**	**H-type**	**C-type**	**H-type**	**C-type**	**H-type**	**C-type**	**H-type**

SAF32	+	-	±	-	-	-	+	±	+	-	±	-	±	-
B103	2+	+	+	±	2+	±	+	+	2+	-	+	-	+	-
F89/160.1.5	2+	2+	2+	2+	2+	2+	3+	3+	3+	2+	2+	+	±	-
T1	2+	+	2+	±	2+	+	2+	3+	2+	2+	2+	+	+	-
12F10	3+	2+	3+	+	3+	+	3+	2+	3+	2+	3+	+	2+	-
SAF54	2+	±	2+	-	2+	-	2+	+	3+	+	2+	+	+	-
44B1	3+	2+	3+	2+	3+	2+	3+	3+	3+	2+	3+	2+	+	+
SAF84	3+	2+	3+	+	3+	3+	3+	3+	3+	3+	3+	2+	2+	-
43C5	3+	2+	3+	+	3+	+	3+	2+	3+	2+	3+	2+	2+	+
T4	3+	2+	3+	2+	3+	2+	3+	3+	3+	3+	3+	3+	+	3+
F99/97.6.1	3+	3+	3+	3+	3+	3+	3+	3+	3+	3+	3+	3+	2+	3+

-, none; ±, negligible; +, weak; 2+, distinct; 3+, strong

### PrP^Sc ^deposition in additional structures

Positive PrP^Sc ^immunolabeling was detected in the trigeminal and dorsal root ganglia, neurohypophysis, retina, and optic nerve. In the neurohypophysis, fine granular PrP^Sc ^depositions were detected in the unmyelinated nerve fibers, and intracytoplasmic immunolabeling was detected in the pituicytes. In the retina, intense granular immunolabeling was observed in the ganglion cell layer as well as the inner and outer plexiform layers. In the optic nerve, intraglial immunolabeling was prominent. PrP^Sc ^was occasionally found in satellite cells and ganglion cells of the trigeminal and dorsal root ganglia. No PrP^Sc ^immunolabeling was detected in the lymphoid tissues, including the spleen, tonsils, Peyer's patches, and lymph nodes, in any of the animals.

### Western blot analysis

**Figure 5:**
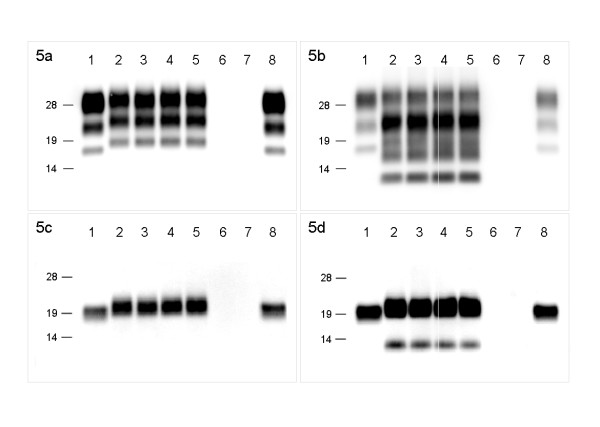
**Western blot analysis**. Western blot analysis of PrP^res ^derived from C-type BSE- and H-type BSE-challenged cattle, with mAbs 6H4 (a and c) and SAF84 (b and d). Lanes 1 and 8, C-type BSE tissue; lanes 6 and 7, PrP^res^-negative bovine brain. Other lanes show banding for H-type BSE tissue: lane 2, Canadian case; lane 3, case 1 (code 7749); lane 4, case 2 (code 9458); and lane 5, case 3 (code 0728). In panel d, the band near 14 kDa is of lower molecular mass than 14 kDa. All the samples were digested with 50-μg/mL PK at 37°C for 1 h, and then treated with PNGase F (c and d). Molecular mass markers (kDa) are shown on the left.

PrP^res ^was detected by western blot analysis using mAbs 6H4 and SAF84 in all the animals challenged with H-type BSE (Figure 5), whereas the control animals were negative for PrP^res^. Western blot analysis with 6H4 showed that the diglycosylated, monoglycosylated, and unglycosylated fragments of PK-treated PrP^res ^derived from H-type BSE-challenged cattle were more than those of PrP^res ^derived from the C-type BSE-affected cattle. On the contrary, the glycoform profiles of PrP^res ^derived from both the H-type BSE- and C-type BSE-challenged cattle were similar (Figure 5a and 5c). With mAb SAF84, a multiple banding pattern was detected from the H-type BSE sample (Figure 5b). After deglycosylation with PNGase F treatment, in addition to ~19 kDa PrP^res ^fragment, a 10-12 kDa PrP^res ^fragment was detected from the H-type BSE sample (Figure 5d). This additional 10-12 kDa fragment was not recognized with 6H4. The molecular size and glycoform patterns of PrP^res ^were similar and conserved in the H-type BSE-challenged cattle.

**Figure 6:**
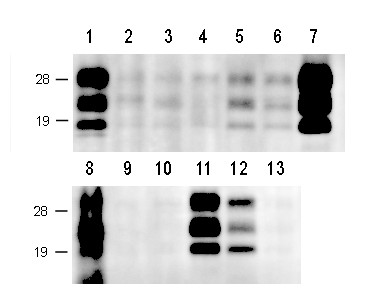
**Western blot analysis of PrP**^**res **^**in nervous tissue**. Western blot analysis of PrP^res ^in the nervous tissue sampled from the H-type BSE-challenged cattle (case 1, code 7749). 1, cranial cervical ganglion; 2, vagal nerve (cervical division); 3, vagal nerve (thoracic division); 4, phrenic nerve; 5, sympathetic trunk (thoracic division); 6, sympathetic trunk (lumbar division); 7, trigeminal ganglion; 8, adrenal gland (medulla); 9, facial nerve; 10, hypoglossal nerve; 11, celiac-mesenteric ganglion complex; 12, cauda equina; 13, suprascapular nerve. Each lane was loaded with 100 mg of tissue equivalent. Western blots were probed with mAb 6H4 to detect PrP^res^. Molecular mass standards (kDa) are indicated on the left.

In addition to the brain and spinal cord, PrP^res ^was also detected in most of the peripheral nerves, ganglia, optic nerve, retina, hypophysis, and adrenal gland. The intensity of the signal from most of these tissues was, however, barely detectable, but the characteristic triple banding was always detected (Figure 6). No PrP^res ^signal was detected in the lymphoid tissues of the three challenged calves. The results of the western blot analysis are summarized in Table 3.

**Table 3:** Immunohistochemical and western blot analyses of PrP^Sc ^in tissue samples obtained from cattle intracerebrally challenged with H-type BSE

Tissue	Case code					
	
	7749		9458		0728	
	
	IHC	WB	IHC	WB	IHC	WB
Central nervous system						
Cerebral cortex	+	+	+	+	+	+
Obex	+	+	+	+	+	+
Cerebrum	+	+	+	+	+	+
Spinal cord	+	+	+	+	+	+
Peripheral nervous system						
Cauda equina	+	+	+	+	+	+
Dorsal root ganglia	+	+	+	+	+	+
Trigeminal ganglia	+	+	+	+	+	+
Cranial cervical ganglia	-	+	-	+	-	+
Stellate ganglia	-	+	-	+	-	+
Sympathetic trunk	-	+	-	+	-	+
Celiac-mesenteric ganglion complex	-	+	-	+	-	+
Vagus nerve	-	+	-	+	-	+
Facial nerve	-	+	-	+	-	-
Hypoglossal nerve	-	+	-	+	-	-
Phrenic nerve	-	+	-	+	-	+
Accessory nerve	-	-	-	+	-	+
Suprascapular nerve	-	+	-	+	-	+
Brachial plexus	-	+	-	+	-	+
Median nerve	-	+	-	+	-	+
Radial nerve	-	+	-	+	-	+
Sciatic nerve	-	+	-	+	-	+
Tibial nerve	-	+	-	+	-	+
Optic nerve	+	+	+	+	+	+
Retina	+	+	+	+	+	+
Pituitary gland	+	+	+	+	+	+
Adrenal gland	-	+	-	+	-	+
Lymphoid tissues						
Spleen	-	-	-	-	-	-
Tonsils (palatine, pharyngeal, lingual)	-	-	-	-	-	-
Thymus	-	-	-	-	-	-
Parotid lymph node	-	-	-	-	-	-
Mandibular lymph node	-	-	-	-	-	-
Lateral retropharyngeal lymph node	-	-	-	-	-	-
Superficial cervical lymph node	-	-	-	-	-	-
Brachiocephalic lymph node	-	-	-	-	-	-
Axillary lymph node	-	-	-	-	-	-
Superficial inguinal lymph node	-	-	-	-	-	-
Subiliac lymph node	-	-	-	-	-	-
Popliteal lymph node	-	-	-	-	-	-
Hepatic lymph node	-	-	-	-	-	-
Internal iliac lymph node	-	-	-	-	-	-
External iliac lymph node	-	-	-	-	-	-
Mesenteric lymph node	-	-	-	-	-	-

IHC, immunohistochemistry; WB, western blotting; +, positive for PrP^Sc^; -, negative for PrP^Sc^

### Analysis of *PRNP*

The mature PrP sequences (amino acids 25-242) of the *PRNP *ORF in the three challenged animals were compared with the representative bovine *PRNP *sequence (GenBank accession number: AJ298878) [37]. The *PRNP *sequence for case 2 (code 9458) was the same as the reference sequence, and that for case 1 (code 7749) was also normal with a synonymous polymorphism at codon 78 (G or A; no amino acid substitution). The 2 animals had 6 copies of the octarepeat region on both the *PRNP *alleles. The *PRNP *sequence for case 3 (code 0728) was also normal, and both alleles contained five copies of the octarepeat region.

## Discussion

This study demonstrated successful intraspecies transmission of H-type BSE characterized by a shorter incubation period as compared with C-type BSE [19]. To the best of our knowledge, thus far, neuropathological and immunohistochemical data for H-type BSE have only been reported from the medulla oblongata at the obex in German, United States, and Swedish field cases [10,13,24]. This is related to the fact that only the obex region is sampled for BSE rapid tests and other brain regions are often unavailable due to marked autolysis, limitations in collection infrastructure, or freezing artifacts [10,13,24,25]. This is the first presentation of H-type lesion profiles involving the whole CNS and additional nervous tissues, although of experimentally infected animals.

Incubation periods in the cattle challenged with the Canadian H-type BSE (mean period, 18 months) were two months longer than those reported in cattle challenged with German H-type BSE [20]. This difference in incubation periods has several potential explanations, which include differences in agents tested, inoculum titers, and breeding conditions. Infectivity titer issues might be resolved by comparing second-passage infection experiment results.

Spongy changes were generally present in the gray matter throughout the brain and spinal cord, but were more conspicuous in the cerebral cortices, thalamus, hypothalamus, and midbrain. In most brain areas, vacuoles were generally detected in the neuropil and only occasionally in the neurons. The spatial distribution pattern of spongiform changes and immunolabeled PrP^Sc ^in the brain of an H-type BSE-infected Zebu, analyzed with N-terminal-specific mAb P4 and C-terminal-specific mAb F99/97.6.1, was similar to that in C-type BSE cases [38]. In natural and experimental C-type BSE cases, spongiform lesions are consistently distributed throughout the brain, but overall, the lesions in the thalamus and brainstem including the midbrain and medulla oblongata at the obex are more severe than those in the cerebral cortices [29,39]. The results of the present study indicate that the vacuolar lesion score of the H-type BSE-challenged cattle was higher than that of C-type BSE-affected cattle [19,29,40,41]. Moreover, the topographical distribution of PrP^Sc ^in the brain of BSE-infected sheep is similar irrespective of the different challenge routes such as intracerebral, intravascular, or intraperitoneal route [42], suggesting common patterns of neuroinvasion and CNS spread [43]. On the contrary, the minor differences detected in the distribution of PrP^Sc ^in the brain between deer that are orally and intracerebrally infected with BSE may be due to differences in the routes of infection [44].

The immunolabeling patterns of PrP^Sc ^in the cattle affected with H-type BSE were characterized by the presence of both PrP^Sc^-positive plaques and intraglial- and stellate-type PrP^Sc ^accumulations in the brain. Severe intraneuronal- and intraglial-type PrP^Sc ^accumulations as well as plaque-like PrP^Sc ^aggregates with the absence of stellate-type PrP^Sc ^deposition have been reported in the obex region of H-type BSE-affected animals [10,13]. These immunohistochemical features were detected in the obex region and coincided with those observed in the present study. However, neither amyloid plaques nor stellate-type PrP^Sc ^depositions have been reported in H-type BSE-affected cattle, most likely due to their limitation to the medulla oblongata at the obex [8,10,13,24].

Two different types of plaques were found in this study: unicentric and multicentric PrP plaques. Most of these plaques were uniformly immunopositive for PrP, with a dense non-Congophilic core. The plaques that had a pale central core with a Congophilic reaction were less frequent. It has been suggested that Congophilic plaques may correspond with the late stage of plaque formation, whereas non-Congophilic plaques coincide with the early stage of CJD and Gerstmann-Sträussler-Scheinker syndrome [45]. The 2 types of PrP^Sc^-positive plaques--unicentric and multicentric--have been described in L-type BSE [5,19,46]. Our results indicate that the presence of PrP^Sc ^plaques in the forebrain but not in the brainstem is one of the neuropathological features in cattle affected with atypical BSE. In addition, glial-type PrP^Sc ^deposition in the white matter throughout the brain seems to be a characteristic feature of H-type BSE in cattle, as supported by identical findings in German and Swedish H-type BSE field cases [10,13].

Extracellular PrP^Sc ^was immunolabeled with N-terminal-, core-, and C-terminal-specific antibodies, but intracellular PrP^Sc ^did not show immunoreactivity to the N-terminal-specific anti-PrP antibodies [47,48]. Intracellular PrP^Sc ^has markedly diminished immunoreactivity to N-terminal-specific anti-PrP antibodies [47]. However, N-terminal-specific mAb P4, which recognizes an epitope at bovine PrP residues 101-107, showed intraneuronal PrP^Sc ^immunolabeling in sheep affected with C-type BSE [47] and in Zebu affected with H-type BSE [38]. These results indicate that the epitope region for either mAb P4 or core-specific anti-PrP antibodies is located upstream of an intracellular truncation site [38,48]. The differences in intracellular PrP^Sc ^truncation sites between sheep scrapie and ovine BSE [47] as well as between C-type BSE and H-type BSE [38] most probably depend on the strain and the tissues and cells [47]. The intensity and patterns of PrP^Sc ^immunolabeling varied with the different anti-PrP antibodies used, and the difference in the PrP^Sc ^immunohistochemical labeling results might be related to the application of different technical protocols, especially antigen retrieval methods [49-51].

The western blot profiles of PrP^res ^for the H-type BSE-challenged cattle and the Canadian H-type BSE-infected brain homogenate used as inoculum were indistinguishable. Results of previous studies prove that H-type BSE isolates have distinct biological and biochemical properties compared with C-type and L-type BSE isolates [3,52,53]. The PrP^res ^in H-type BSE, as detected by mAb SAF84 recognizing the C-terminus of PrP, was thought to be composed of 2 fragments with molecular masses of 19 kDa and 10-12 kDa, possessing a different cleavage site in the N-terminal region with PK digestion [53]. The higher molecular mass of the unglycosylated PrP^res ^molecules, which included an additional 10-12 kDa fragment, in the Canadian H-type BSE case was maintained in the challenged animals. These unique molecular features of PrP in H-type BSE are also well preserved in transgenic and wild type mice [16,53]. In addition, a distinct 10-12 kDa fragment detected with C-terminal-specific antibodies in H-type BSE might be associated with the presence of PrP plaques [53].

Although PrP^C ^glycosylation seems to play a critical role in the maintenance of strain-dependent prion neurotropism [54,55], a recent study has demonstrated that PrP^Sc ^glycosylation is not required for the maintenance of strain-specific neurotropisms [56]. Strain-dependent prion neurotropism is currently unknown, but several possibilities have been indicated [56]. Moreover, a local difference in the PrP^Sc ^replication rate may be attributed to a high degree of neurotropism in H-type BSE similar to that observed in C-type BSE [57].

Since 2003, sporadic and discontinuous occurrence of atypical BSE has been detected in Europe, North America, and Japan. Although, till date, the origin and frequency of atypical BSE is unknown, a high prevalence is found in older cattle over the age of eight years. This is the result of the active surveillance programs using rapid screening tests, with the exception of a Zebu case [38]. It has been reported that H-type BSE can be the result of a naturally occurring, heritable variant caused by glutamic acid/lysine polymorphism at codon 211 of the bovine *PRNP *gene (E211K) [11,58]. However, our cases, although experimentally challenged via the intracranial route, and the original Canadian H-type BSE field case [11,58] developed the disease without the novel mutation E211K within *PRNP*. Therefore, atypical BSE seemed to be sporadic rather than inherited with a higher risk in fallen stock than in healthy slaughtered cattle [8,13,25], suggesting that young adult cattle affected with atypical BSE might be dormant carriers. Further studies are required to determine the epidemiological significance and origin of atypical BSE.

The present study demonstrated successful intraspecies transmission of H-type BSE to cattle and the distribution and immunolabeling patterns of PrP^Sc ^in the brain of the H-type BSE-challenged cattle. TSE agent virulence can be minimally defined by oral transmission of different TSE agents (C-type, L-type, and H-type BSE agents) [59]. Oral transmission studies with H-type BSE-infected cattle have been initiated and are underway to provide information regarding the extent of similarity in the immunohistochemical and molecular features before and after transmission. In addition, the present data will support risk assessments in some peripheral tissues derived from cattle affected with H-type BSE.

## Competing interests

The authors declare that they have no competing interests.

## Authors' contributions

Conception and design of experiments: TY and HO. Conduction of experiments: HO, YI, MI, KM, and YM. Intracerebral inoculation of H-type BSE isolate and collection of samples from H-type BSE-infected cattle: YI, HO, MI, KM, YS, and KK. Manuscript draft preparation and data analysis: HO, YI, MI, and YM. Participation in scientific discussion of the results: SC. Study supervision: SM. All the authors have read and approved the final manuscript.
